# Recombinant Antigen for Newcastle Disease Virus Vaccine Focusing on Currently Circulating Virus Variants

**DOI:** 10.3390/ijms27198673

**Published:** 2026-09-28

**Authors:** Elizaveta P. Umarova, Ekaterina M. Ryabchevskaya, Dmitriy L. Granovskiy, Ekaterina A. Evtushenko, Angelina O. Kovalenko, Elizaveta Yu. Boravleva, Alexandra S. Gambaryan, Irina N. Matveeva, Nikolai A. Nikitin, Olga V. Karpova

**Affiliations:** 1 Department of Virology, Faculty of Biology, Lomonosov Moscow State University, 119234 Moscow, Russia; umarovaep@my.msu.ru (E.P.U.); granovskiydl@my.msu.ru (D.L.G.); evtushenkoea@my.msu.ru (E.A.E.); angelina-kovalenko94@mail.ru (A.O.K.); nikitin@mail.bio.msu.ru (N.A.N.); karpovaov@my.msu.ru (O.V.K.); 2 Chumakov Federal Scientific Center for Research and Development of Immune and Biological Products, Russian Academy of Sciences, 108819 Moscow, Russia; elisavetbor@gmail.com (E.Y.B.); al.gambaryan@gmail.com (A.S.G.); 3 All-Russian Scientific Research and Technological Institute of Biological Industry, 17, Biocombinat, 141142 Moscow, Russia; biolog1967@mail.ru

**Keywords:** Newcastle disease virus, recombinant antigen, Newcastle disease virus genotype VII

## Abstract

Newcastle disease virus (NDV) causes a highly contagious avian infection and is characterised by its high genetic diversity and mutation rate. Currently applied vaccines are based on NDV genotypes I and II, which are considerably distinct from the circulating pathogenic genotypes, of which genotype VII is predominant, and therefore, they may not provide complete protection for birds or stop virus transmission. This work is a proof-of-concept study aiming to develop a genotype VII-focused recombinant antigen for a vaccine targeting relevant NDV strains. The research primarily focuses on novel antigen design based on the bioinformatics analysis of recent circulating isolates. Haemagglutinin-neuraminidase (HN) is one of the two main NDV antigens. Two HN fragments (“1”—341–368 aa and “2”—239–257 aa) were selected due to the presence of linear neutralising epitopes. An approach focusing on using a number of selected-fragment variants simultaneously was applied for the first time. The resulting antigen Castle1 encompasses the sequence diversity of most circulating isolates. Preliminary immunological evaluation showed that antibodies from Castle1-immunised animals possessed haemagglutination inhibition activity and interacted with NDV of genotypes I and VII. That makes Castle1 a promising antigen for the further development of a vaccine for preventing ongoing and incoming NDV outbreaks.

## 1. Introduction

Newcastle disease virus (NDV) is a causative agent of a highly contagious avian disease of wild and domestic birds, especially chickens. The disease is widespread throughout the world and leads to severe economic damage. Outbreaks occur in countries at any level of economic development. For example, outbreaks were reported in Slovakia, Bulgaria, Latvia, the Czech Republic, and North Macedonia in 2025–2026 [1]. It is worth mentioning the case in France in 2025 when NDV was collected from a human. This human isolate belonged to the genotype VI.2.1.2 (https://www.ncbi.nlm.nih.gov/protein/YCO90810, accessed on 9 July 2026).

NDV belongs to the *Orthoavulavirus* genus (subfamily *Avulavirinae*, *family Paramyxoviridae*) and is designated as *Orthoavulavirus javaense* according to the International Committee on Taxonomy of Viruses. NDV is an enveloped virus with non-segmented negative-sense genomic RNA. Virions are pleomorphic and have a diameter of 100–500 nm. NDV bears two types of surface glycoproteins—haemagglutinin-neuraminidase (HN) and fusion protein (F). Protein F mediates the fusion of virus and host cell membranes. HN provides the activation of the F-protein and binding to cell receptors containing sialic acid and facilitates virus release via sialic acid residue modification. Both of these glycoproteins are antigens of the virus and stimulate the production of neutralising antibodies [2].

NDV tends to accumulate mutations rapidly. There is considerable genetic and antigenic heterogeneity across the viral isolates [3,4,5]. Modern classification is based on the phylogenetic analyses of whole gene F sequences and includes two classes. Class I is represented by a single genotype 1, which is separated into subgenotypes. Class II consists of 20 genotypes, some of which are also divided into subgenotypes [6]. Currently, the most velogenic (highly virulent) NDV strains belong to genotypes V, VI, and VII [7] and lead to a mortality rate of up to 100% in infected birds [8,9].

Currently, live attenuated and inactivated vaccines such as LaSota, B1, VG/GA, and others are used on poultry farms [2]. They are based on genotypes I and II and therefore genetically and antigenically distant from the most prevalent and pathogenic genotype VII. Hence, they cannot protect birds completely and are unable to delay the dissemination of the pathogenic virus [10,11]. Thus, the development of a vaccine with high efficacy especially against currently circulating highly pathogenic NDV is a primary concern.

The development of subunit recombinant vaccines based on HN and F viral antigens stimulating the production of neutralising antibodies is a promising approach due to their high safety profile alongside the simplicity of production. There are subunit vaccine candidates comprising recombinant haemagglutinin-neuraminidase (rHN) and/or recombinant fusion protein (rF) produced in various expression systems. Lee et al. (2008) generated rHN- and rF-proteins (in a baculovirus expression system) which, in combination with an adjuvant, provided complete protection of chickens from clinical Newcastle disease and death [12]. Administration of rHN from a suspension-cultured tobacco cell line provided 90% protection against lethal challenge (the challenge-virus genotype is unmentioned by the authors) [13]. Despite USDA approval, this vaccine was not commercialised. The rHN-protein (C-terminus of HN protein, 256–577 aa, fused with GST) from *E. coli* increased the protection rate of inactivated vaccines against a velogenic strain (Sato, genotype III). However, it was poorly immunogenic by itself and did not elicit noticeable antibody response in chickens [14].

One of the promising directions in recombinant subunit vaccine development is the development of epitope vaccines [2,15,16]. As far as NDV major antigens are concerned, it has been shown for the F-protein that its epitopes are conformational and depend on the correct protein folding [17,18]. On the contrary, the HN protein of NDV contains at least five linear epitopes that were tested by immunochemical methods, and for at least two of them, neutralising activity was confirmed [3,4,19,20,21,22]. This makes HN more preferable for the development of an epitope vaccine.

In the present work, a new recombinant antigen consisting of HN antigenic fragments—Castle1—was designed. A comprehensive analysis of relevant NDV isolates collected worldwide after 2015 was conducted in the current study to select the appropriate sequences of HN fragments to include in Castle1. Castle1 was synthesised in an *E. coli* expression system. Its antigenic properties, including the ability to induce the production of NDV-binding antibodies with haemagglutination inhibition activity of the virus in vitro, were examined.

## 2. Results

### 2.1. Recombinant HN-Based NDV Antigen Design

**Figure 1:**
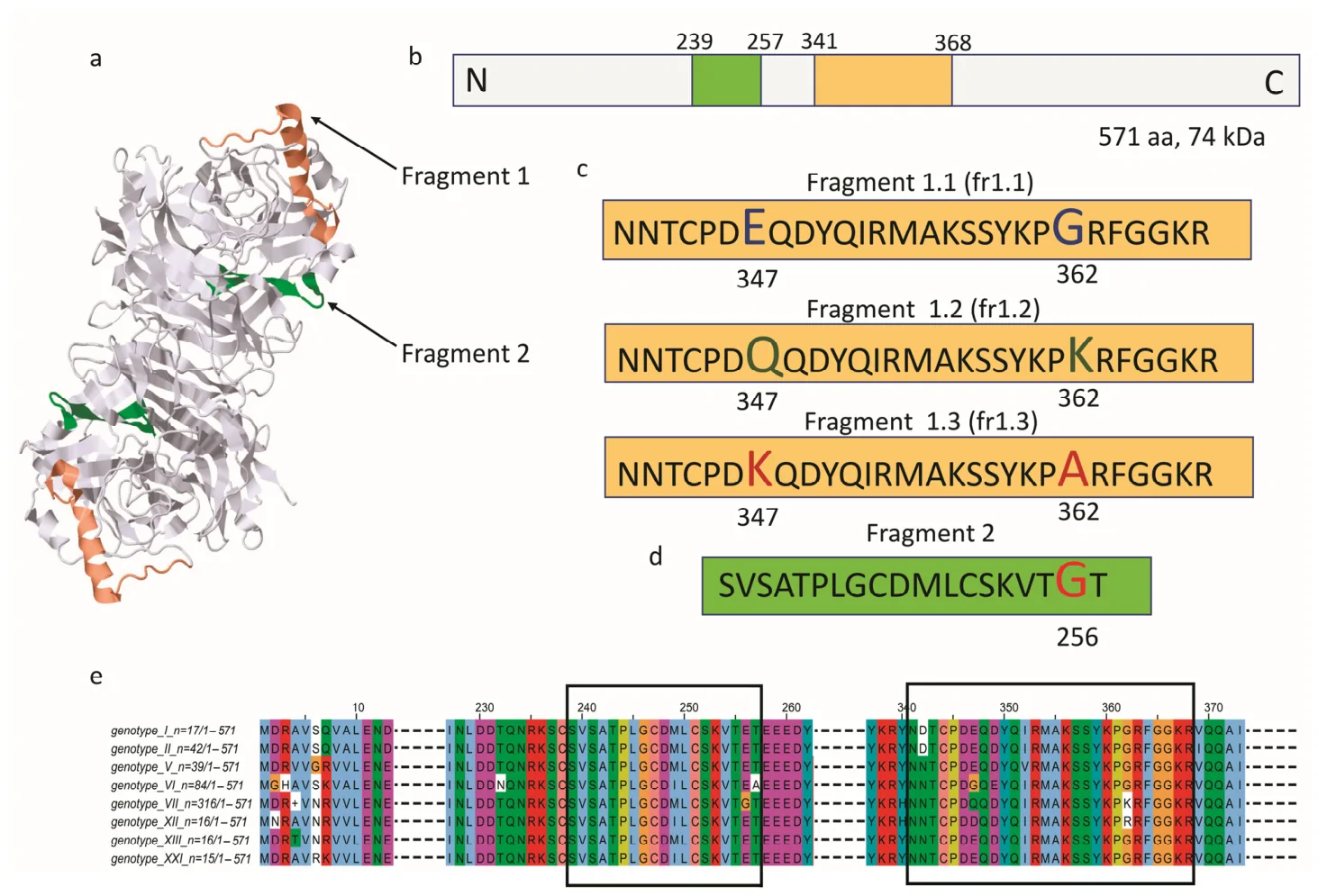
The selected epitopes and their location within the haemagglutinin-neuraminidase structure. (**a**) Haemagglutinin-neuraminidase model image (Protein Data Bank [PDB]: 1USR [26,27]. (**b**) Linear diagram of the NDV HN protein (1–571 aa) showing the location of fragments 1 (341–368 aa) and 2 (239–257 aa) (not to scale). (**c**) Variants of amino acid sequences of fragment 1 (341–368 aa) of the Castle1 antigen. (**d**) Amino acid sequence of fragment 2 (239–257 aa) of the Castle1 antigen. (**e**) Comparison of the consensus sequences of the HN gene fragments 341–368 aa and 239–256 aa. The number of sequences used for designing each genotype consensus is indicated in brackets (only sequences with collection dates after 2015 were used). Selected epitopes are distinguished by boxes.

Here, the HN-based recombinant antigen Castle1 was designed. We selected two fragments corresponding to amino acids 341–368 (fragment 1, fr1) and 239–257 (fragment 2, fr2), which contain neutralising epitopes of HN, as a basis for Castle1. The selection of the fragments was based on published experimental and *in silico* studies [3,22,23,24,25]. The location of the selected fragments in the HN protein model and linear diagram is represented in Figure 1a,b, respectively.

The heterogeneity of the chosen HN sequences among modern NDV isolates was taken into account. For this, we analysed NDV isolates from GenBank with known genotypes and collection dates between 2015 and 2025. The list of sequences included in the analysis is presented in Supplementary Table S1. The genotype distribution is shown in Table 1. Class I of NDV has minimal virulence in chickens [2] and was therefore beyond the scope of our study. The consensus sequences were built for genotypes I, II, V, VI, VII, XII, XIII, and XXI of class II. Genotypes III and IV were not included in further analyses because only two sequences with relevant collection dates were found for each of them. The comparison of the consensus sequences of fr1 and fr2 is presented in Figure 1e. The majority of the NDV sequences collected within 2015–2025 belong to the highly pathogenic genotype VII. Therefore, to address the effective prevention of currently circulating highly pathogenic NDV, we were primarily focused on genotype VII. Fr1 and fr2 sequences were built based on the genotype VII consensus.

However, even within isolates belonging to genotype VII, high heterogeneity in 347 and 362 amino acids (fr1) was revealed (Supplementary Table S2). The substitutions in the position 347 are known to dramatically change the antigenic properties of HN [3,4,19,20,21]. At the same time, the variations at positions 347 and 362 are often covariant [4,19]. We calculated the frequency of occurrence of different 347/362 pairs among all sequences used for consensus preparation (Supplementary Table S2). Undoubtedly, the genotype VII data were of primary interest to us. To expand the coverage of virus variants, three sequences corresponding to the most frequent pairs among genotype VII isolates were included in the designed recombinant protein: variant 1 (fr1.1) contains 347E/362G, variant 2 (fr1.2) contains 347K/362A, and variant 3 (fr1.3) contains 347Q/362K (Figure 1c). Notably, the first variant 347E/362G is also the most common for genotypes I, II, V, and XXI. Genotypes I and II are lentogenic and include vaccine stains Bor-74 and LaSota, respectively. Genotypes V and XXI are pathogenic.

**Figure 2:**
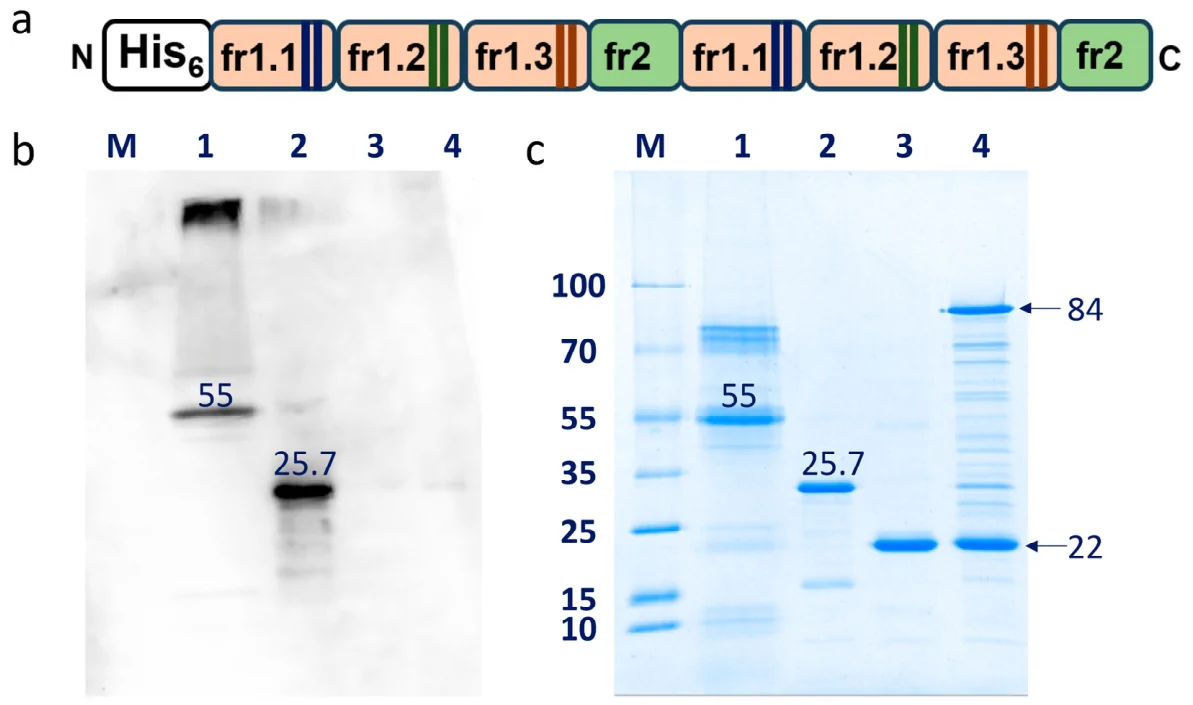
Castle1 antigen and its interaction with polyclonal serum to NDV. (a) Castle1 antigen schematic representation (not to scale). The fragments include the following amino acid sequences corresponding to the most frequent HN sequences among genotype VII modern isolates: fr1.1— ^341^NNTSPD**E**QDYQIRMAKSSYKP**G**RFGGKR^368^, fr1.2— ^341^NNTSPD**K**QDYQIRMAKSSYKP**A**RFGGKR^368^, fr1.3— ^341^NNTSPD**Q**QDYQIRMAKSSYKP**K**RFGGKR^368^, and fr2—^239^SVSATPLGSDMLSSKVTGT^257^. Colours (blue, green, and brown) indicate variable aa in fr1.1, fr1.2, and fr1.3 respectively. (**b**) Western blot analysis with mouse serum to NDV_Bor-74_ (1:5000) as primary antibodies and secondary HRP-conjugated antibodies (1:10,000). (**c**) Electrophoresis analysis in 8–20% SDS-PAGE, stained by Coomassie G-250. 1—NDV_Bor-74_ (positive control), 2—Castle1, 3—recombinant His_6_-containing coronavirus antigen* expressed in *E. coli* (negative control), 4—Alternanthera mosaic virus + *E. coli*-derived recombinant His_6_-containing protective antigen of *Bacillus anthracis* ** (negative controls). Protein ladder molecular weights in kDa are indicated. *—Obtained previously in Kovalenko et al., 2022 [28]. **—Obtained previously in Ryabchevskaya et al., 2022 [29].

Fragment 2 is quite conservative (Figure 1d), and the simultaneous presence of three fragment 1 variants improves antigenic coverage of the designed protein. The resulting gene containing a duplicated fr1.1-fr1.2-fr1.3-fr2 sequence was cloned into the pQE-30 expression vector. Thus, the generated antigen, which was named Castle1, contained two copies of each variant of the fr1 and fr2 fragments, and the N-terminal His_6_-tag (Figure 2a).

### 2.2. Castle1 Expression and Characterisation

Expression of the Castle1 protein was performed in two strains of *E. coli* cells: XL1-Blue and SG13009. Both strains provided high protein accumulation after induction (Supplementary Figure S1). The molecular weight of Castle1, calculated by amino acid sequence via the ProtParam EXPaSy proteomics server (Swiss Institute of Bioinformatics, http://expasy.org/, accessed on 9 July 2026), is 25.8 kDa, which is consistent with that revealed by SDS-PAGE analysis (Figure 2c, lane 2).

Castle1 was recognised by polyclonal mouse serum specific to the NDV_Bor-74_ strain (Figure 2b, lane 2), which also interacted with the positive control NDV_Bor-74_ (Figure 2b, lane 1). Two different His_6_-tag-containing recombinant proteins (coronavirus antigen and protective antigen of *Bacillus anthracis*), were used as negative controls (Figure 2c, lane 3 and lane 4 upper band, respectively). Alternanthera mosaic virus was used as an additional non-recombinant control (Figure 2c, lane 4 lower band). None of the controls interacted with anti-NDV_Bor-74_ serum; this proves the specificity of its interaction with Castle1.

### 2.3. Poultry Farm Chicken Sera Characterisation

For further use in the present study, poultry farm chicken sera were analysed in ELISA. Antibody titres to two different NDV isolates—NDV_6081_ (NDV/chicken/Moscow/6081/2022, genotype VII) and NDV_3639_ (NDV/duck/Moscow/3639/2008, genotype I)—were assessed. A group of sera (*n* = 9) collected from non-vaccinated SPF chickens was used as a non-immune control. Anti-NDV_3639_ and anti-NDV_6081_ IgY titres are presented in Supplementary Figures S2a and S2b, respectively. Both anti-NDV_3639_ and anti-NDV_6081_ IgY titres were significantly higher in poultry farm chicken sera comparing to those in the non-immune control group. Therefore, the analysed poultry farm chicken sera were proved to be immune to NDV.

### 2.4. Castle1 Interacts with the Serum of Chickens Immune to NDV

**Figure 3:**
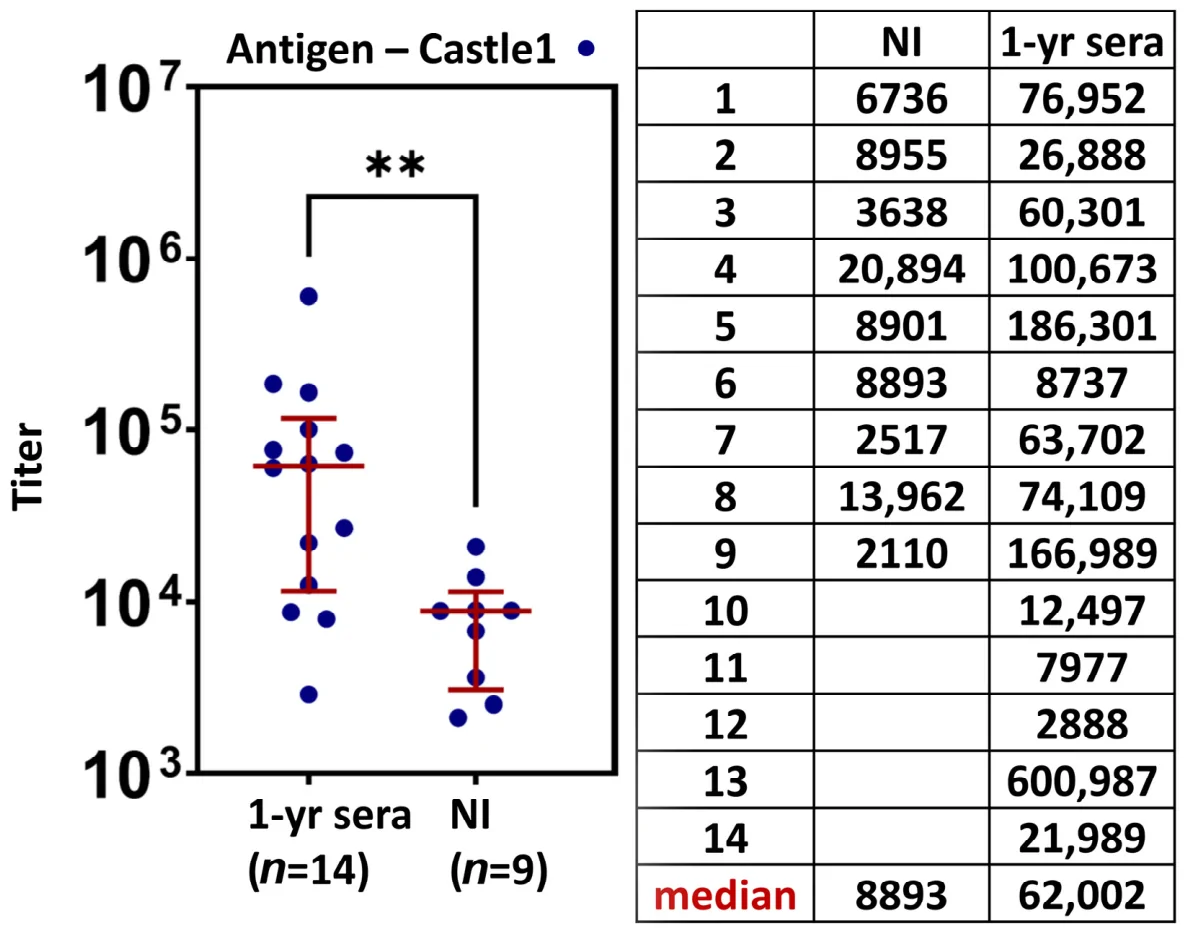
Castle1 efficiently interacts with sera from the poultry farm. Sera titres were evaluated using indirect ELISA. The group of poultry farm sera was compared with the non-immune group. *p*-values were calculated using the Mann–Whitney Test. ●, IgY titres of individual chickens; 1 yr sera, 1-year-old chicken sera group; NI, non-immunised group; ** *p* < 0.01; ▬, median. Error bars represent the interquartile range.

The interaction of Castle1 with sera of chickens from a poultry farm immune to NDV was evaluated. Anti-Castle1 titres of immune and control (from intact non-immune SPF chickens) sera are presented in Figure 3. The titres in the immune group of sera (median = 62,002) were significantly higher than those in the control group (median = 8893). This result shows that IgY in poultry farm chicken sera reliably and specifically recognise Castle1.

### 2.5. Characterisation of the Antigenic Diversity of Fragments Comprising Castle1

To characterise the contribution of each fragment to the Castle1–serum interaction, four recombinant proteins, each containing only one of the fragments fr1.1, fr1.2, fr1.3, or fr2, were obtained. Each fragment was fused to the C-terminus of mouse dihydrofolate reductase (DHFR), which served as a carrier. DHFR also had a His_6_-tag at the N-terminus for purification. DHFR-fr1.1, DHFR-fr1.2, DHFR-fr1.3, and DHFR-fr2 were expressed and purified in the same manner as Castle1 and used to study the antigenic heterogeneity of the fragments by Western blot analysis.

In addition to the previously described 1-year-old (1 yr) chicken sera group, we received 28 sera collected from 2-day-old (2 d) chickens (*n* = 14) and 7-month-old (7 m) chickens (*n* = 14). Among all 42 sera samples, six sera with the highest titres to Castle1 were selected for the following Western blot analysis.

**Figure 4:**
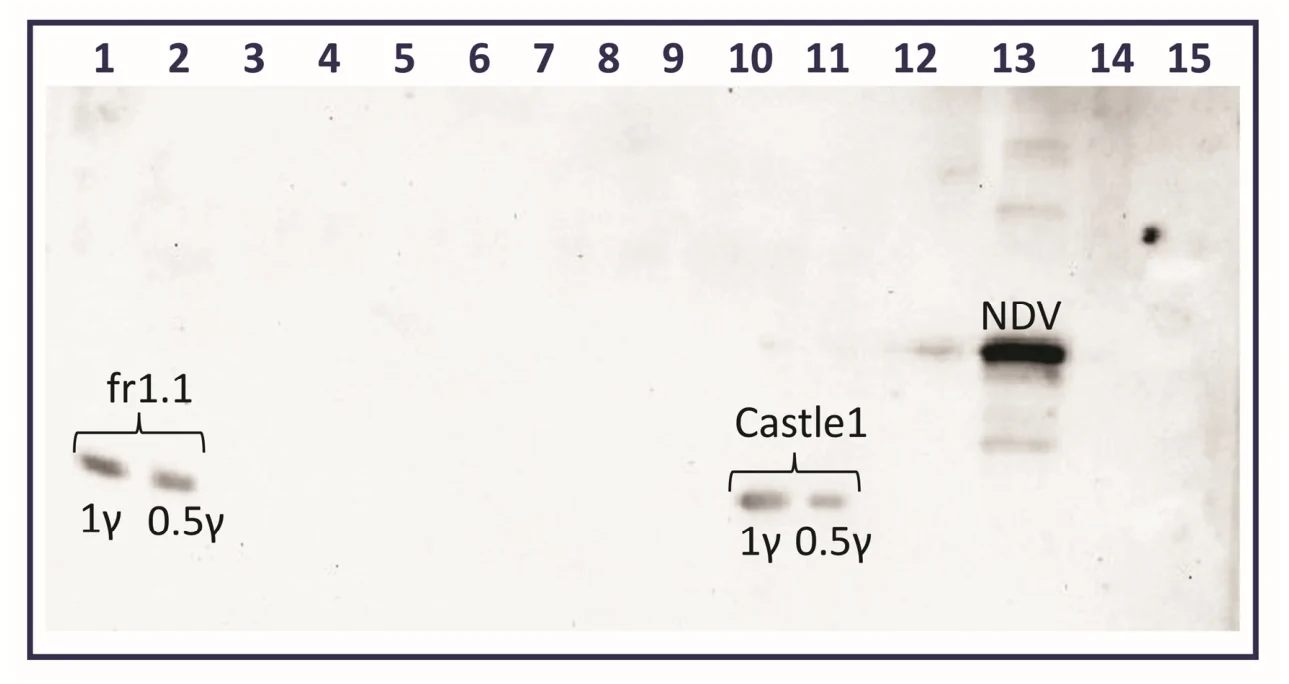
Study of the antigenic diversity of Castle1-composing fragments. Western blot analysis with primary chicken’s serum from poultry farm (1:3000) and secondary HRP-conjugated antibodies (1:10,000). 1 (1γ), 2 (0.5γ)—fr1.1; 3 (1γ), 4 (0.5γ)—fr1.2; 5 (1γ), 6 (0.5γ)—fr1.3; 7 (1γ), 8 (0.5γ)—fr2; 9 (1γ)—DHFR (negative control); 10 (1γ), 11 (0.5γ)—Castle1; 13—NDV (strain Bor-74) (positive control); 14 (1γ)—recombinant His_6_-containing coronavirus antigen* expressed in *E. coli* (negative control); 15—protein MW ladder. The quantity of protein loaded into a slot is indicated: 1γ = 1 μg; 0.5γ = 0.5 μg. *—Obtained previously in Kovalenko et al., 2022 [28].

For four sera (1 yr #13, 7 m #6, 1 yr #9, and 2 d #1), identical results were detected. A representative image is shown in Figure 4 (other blot images are presented in Supplementary Figure S3). These sera clearly recognised Castle1 (Figure 4, lanes 10 and 11) and DHFR-fr1.1 (Figure 4, lanes 1 and 2) with no detectable interaction with the proteins DHFR-fr1.2 (Figure 4, lanes 3 and 4), DHFR-fr1.3 (Figure 4, lanes 5 and 6), or DHFR-fr2 (Figure 4, lanes 7 and 8). To verify the specificity of potential serum–fragment interaction, non-fused DHFR was used as an additional negative control and was shown to be unrecognised (Figure 4, lane 9), along with a foreign coronavirus antigen (Figure 4, lane 14).

Serum 1 yr #5 (Supplementary Figure S4A) did not react with any samples besides NDV_Bor-74_ was used as a positive control. Despite clear recognition under native conditions of ELISA, this serum did not interact with Castle1 under the denaturing conditions of Western blot.

An unexpected result was revealed for serum 7 m #3 (Supplementary Figure S4B): it recognised Castle1 strongly but did not interact with any of the Castle1 fragments fused to DHFR.

Anti-NDV_6081_ and anti-NDV_3639_ IgY titres of additional sera used for Western blot analysis were also identified. Table 2 includes summary data of the ELISA titres and Western blot results. DHFR-fr1.2, DHFR-fr1.3, and DHFR-fr2 are not represented within the table because no tested serum interacted with them under the denaturing conditions of Western blot.

It should be taken into account that the results under native and denaturing conditions could differ. It was found that fragment 1.1 makes the greatest contribution to the interaction of Castle1 with poultry farm chicken sera. This is consistent with the fact that the sequence of fr1.1 coincides with sequences of vaccines used for vaccination of chickens on the farm that provided us with the sera used in the analyses (VECTORMUNE ND, genotype I; Nobilis^®^ ND C2, genotype II; VH vaccine, genotype II).

### 2.6. Antibodies Elicited by Castle1 in Mice Bound NDV

**Figure 5:**
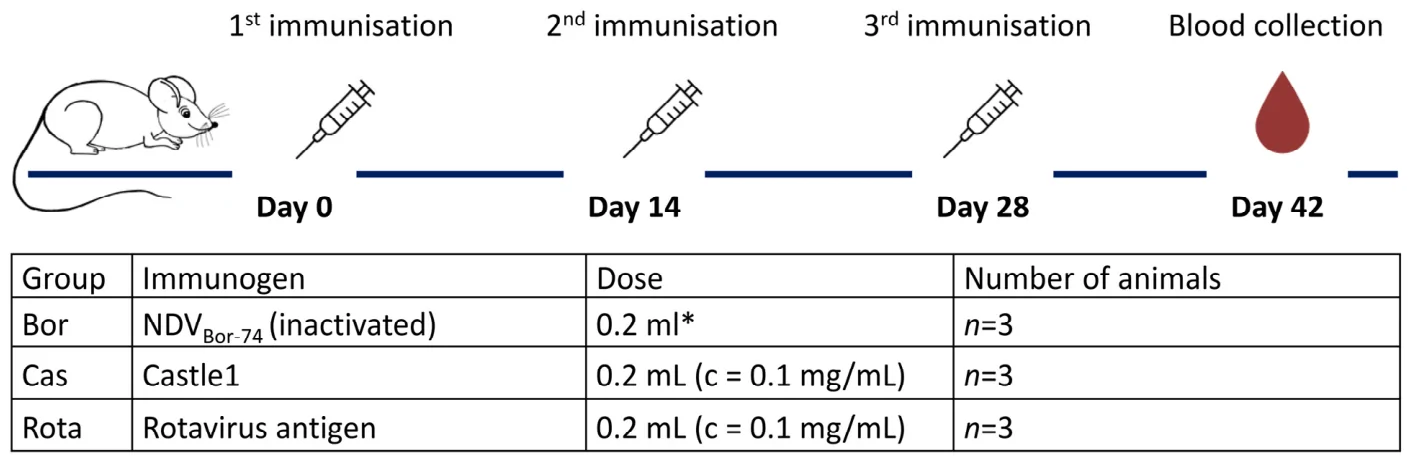
The schedule of mice immunisation. Mice were immunised intraperitoneally with an inactivated sample of NDV_Bor-74_ (0.2 mL*) or with 20 µg of Castle1 or recombinant rotavirus protein** (negative control). All samples were mixed with an equal amount of Freund’s adjuvant (primary immunisation—complete, boosters—incomplete); the final administered volume was 0.4 mL. *—Haemagglutination activity determined before inactivation—9.5 HAU. **—Obtained previously in Granovskiy et al., 2024 [30].

Castle1’s ability to not only bind anti-NDV antibodies but also elicit NDV-binding antibodies by itself was tested. For this purpose, mice were immunised with Castle1. Alongside the group immunised with the Castle1 (“Cas”), the group “Bor” and the group “Rota” were immunised with inactivated NDV_Bor-74_ and a heterologous His_6_-containing recombinant protein based on a rotavirus spike protein sequence (obtained previously in Granovskiy et al., 2024 [30]), respectively. The latter two groups served as positive and negative controls, respectively. The scheme of immunisation and a detailed group description are presented in Figure 5.

The titres of antibodies specific to the Castle1, NDV_Bor-74_, and rotavirus protein antigens, as well as titres to NDV_3639_ and NDV_6081_, were assessed by ELISA. The final titres are presented in Table 3.

All tested sera from groups Bor and Cas had clear specificity to each of the four NDV antigens—Castle1, NDV_Bor-74_, NDV_3639_, and NDV_6081_—and had negligible (interpretated as non-immune) titres to heterologous rotavirus protein. At the same time, sera from the parallel control group Rota strongly bound the rotavirus protein and reacted with none of the NDV antigens. This confirms the specificity of recognition of NDV antigens by the mice sera obtained after immunisation with Castle1. High anti-Castle1 titres of sera from the Bor group additionally confirm the results revealed in Section 2.2 of the Results: sera immune to NDV effectively interact with Castle1 as well.

Even more importantly, high titres of NDV-specific antibodies determined in the sera from the Cas group clearly prove the ability of Castle1 to induce the production of antibodies binding NDV of genotypes I and VII.

Additionally, a pilot experiment addressing the preliminary study of Castle1 immunogenicity in a natural host was carried out on SPF chickens. Sera from chickens were titrated using NDV_3639_ and NDV_6081_ as antigens. Titres of antibodies specific to both antigens were significantly higher in sera from Castle1-immunised chickens in comparison to sera from intact non-immune birds (Supplementary Figure S5).

### 2.7. Antibodies Elicited by Castle1 in Mice Demonstrate Haemagglutination Inhibition Activity

Haemagglutination inhibition (HAI) activity of these sera was analysed. The analysis was performed in three analytical replicates. The mean value of HAI titres of each serum is presented in Table 4. Negative control sera from mice immunised with heterologous His_6_-tag-containing recombinant proteins or tobacco mosaic virus did not demonstrate haemagglutination inhibition activity in the test. At the same time, sera from mice immunised with Castle1 provided a reliable level of haemagglutination inhibition activity that was comparable to the HAI activity level of sera from NDV_Bor-74_-immunised mice.

## 3. Discussion

The aim of the current proof-of-concept study was to design a recombinant NDV antigen targeting currently circulating genotype VII variants, with the potential to broaden antigenic coverage of prevalent NDV variants. Therefore, the first task was to select appropriate linear epitopes.

There are at least two linear epitopes within 341–375 aa of HN [3,4,19,20,21,22,23]. ^346^DEQDYQIR^353^, the first identified linear epitope of HN, interacts in a Western blot with multiple monoclonal antibodies capable of inhibiting the haemagglutination activity of different NDV strains [3]. Also, a peptide corresponding to 341–355 aa induced antibodies with partial virus-neutralising activity against the avirulent LaSota strain [22]. Thus, the epitope located over 341–355 aa is considered neutralising. Furthermore, antibodies to the HN epitope ^355^AKSSYKPGAFGGKRIGGAILS^375^ reacted with NDV of more than twenty different strains (including highly pathogenic genotypes VI and VII) in a Western blot [23]. These experimental data are supported by *in silico* studies. The analysis of amino acid substitutions and bioinformatic epitope prediction suggest the 341–368 aa fragment to contain two linear epitopes promising for vaccine development [19,24,25]. A molecular docking analysis for VIIj and LaSota isolates interacting with MHC I and II molecules revealed seven predicted B-cell or/and T-cell epitopes, including 341–350 aa and 349–367aa [24]. The BepiPred server predicted 321–364 aa to be an HN epitope [25,31]. According to the ElliPro server prediction, 337–357 aa and 360–368 aa form distinct epitopes [25]. Here, considering all previously published results, we selected the 341–368 aa sequence as the first fragment for recombinant antigen design (fragment 1, fr1).

In addition, 242–256 aa was described as a conservative linear epitope, which showed promising results in an experiment of virus neutralisation with two different NDV strains (LaSota—genotype II, JS17—genotype VII) [22]. Therefore, in the current research, fragment 2, which includes 239–257 aa (fr2), was also utilised.

In several works, fragments close in composition to our fr1 and fr2 were designed and expressed in *E. coli*. In a work by Ji et al. (2025), three HN epitopes (genotype VII) including 341–346 aa were assessed. An antigen based on these epitopes was recognised by polyclonal antibodies to NDV in a Western blot and ELISA [32], but there is no way to evaluate the contribution of each certain epitope to antibody binding. Also, the authors mentioned the 355–370 aa and 345–360 aa regions, which are almost completely covered by fr1, as promising epitopes, but their properties were not assessed [32]. In other works, recombinant antigens comprising LaSota-based HN fragments partially overlapping with ours were studied. rHN proteins consisting of 25–100 aa and 243–481 aa [33] or 296–366 aa [34] of HN were recognised by anti-NDV_B1_ sera in a Western blot and ELISA. More importantly, antibodies elicited by the fragment containing 25–100 aa and 243–481 aa were also shown to interact with NDV_B1_ under the native conditions of ELISA [33]. These studies support the selection of fr1 and fr2 in the current research. However, these previously published works did not address the problem of the high mutation rate of NDV leading to increasing antigenic variability.

The following assignment was to determine the exact sequences of the selected fragments that would provide the production of antibodies binding currently circulating NDV variants. To address this assignment, we performed a comprehensive analysis of HN sequences that had been deposited in GenBank during the past decade and built consensus sequences for eight primarily circulating genotypes. Our analysis of isolates collected worldwide in 2015–2025 revealed an actual NDV genotypes distribution and confirmed that genotype VII isolates currently prevail worldwide. That is why the genotype VII modern consensus sequence was used as a basis for recombinant antigen design. However, some amino acid residues (to the greatest extent, 347 and 362) within our selected fragments were revealed to be highly variable even within the certain genotype. Many publications addressed the issue of high variability at position 347 of HN. The substitution of glutamic acid (E) (or glycine (G)) with lysine (K) led to a considerable change in the antigenic properties of the 345–353 a.a. epitope [3,19,20,21]. In 2017, it was reported that two new virulent subgenotype VIIj isolates (Mazandaran and Behshahr) have glutamine (Q) at position 347. The authors believe that the E347Q and G362K substitutions would play a key role in the induction of the host’s humoral immunity [24].

Importantly, the analysis conducted in the present work shows that nowadays, the largest proportion of genotype VII isolates (45% of all isolates with collection dates between 2015 and 2025) have glutamine (Q) at position 347. No less importantly, all of these isolates have lysine (K) at position 362. So, these substitutions are strongly co-variative. Apparently, 347Q/362K isolates belong to the VII-L subgenotype. Notably, although in 2019, HN sequences with the 347Q/362K pair represented a minority among the genotype VII isolates, they have spread globally over the past 5 years and currently dominate among genotype VII isolates worldwide. All genotype VII isolates collected in the Russian Federation since 2015 have the 347Q/362K pair in their HN sequences. Therefore, fragment 1 was included in Castle1 in three variants (fr1.1, fr1.2, and fr1.3—with 347E/362G, 347K/362A, and 347Q/362K pairs, respectively), which represent the most common sequences of modern genotype VII isolates (Figure 1c). Notably, fr1.1 (with 347E/362G) is also the major variant for genotype I, II, V, and XXI isolates (Table S2). This approach provides a broader spectrum of antigenic coverage and has been applied here for the first time to an HN-based NDV recombinant antigen.

As far as fragment 2 is concerned, according to Jin et al. (2021), the ^242^ATPLGSDMLSSKVTE^256^ sequence is highly conserved among genotype I–XIII strains [22]. However, for genotype VII strains, glycine (G) is the most common amino acid at position 256 [22]. In a work by Chang et al. (2023), the E256G mutation was also defined to be inherent to genotype VII NDV [35]. This is completely correlated with the results of our analysis. Thus, the 239–257 sequence corresponding to the consensus sequence of modern genotype VII isolates was applied within the present work (Figure 1d).

While there are NDV vaccine candidates containing various parts of the HN protein overlapping with the fragments selected in the current study [32,33,34], they tend to be based on the LaSota NDV strain (genotype II). The use of LaSota as an only sequence source for antigen design does not allow broad-spectrum prevention of the disease to be provided. Thus, these developments have the same disadvantages as licenced NDV vaccines. The approach to antigen design proposed in our research takes relevant circulating strains into account and therefore has the potential to improve the effectiveness of the resulting antigen for ND prevention. Moreover, its flexibility might be useful in the accelerated development of updated vaccine antigen variants in response to potential changes in circulating NDV genotypes distribution.

There are data indicating that tandem repeats of epitopes can improve their immunogenicity. The titre of induced antibodies can be improved by increasing the number of repeats [36]. Positive experiences with epitope-based recombinant antigens containing tandem repeats of epitopes are described in scientific publications [37,38,39]. For example, Tarigan and co-authors (2023) obtained antigenically and immunogenically active epitopes of the SARS-CoV-2 spike protein represented by four tandem repeats [39]. Considering this, Castle1 was designed as a duplicated fr1.1-fr1.2-fr1.3-fr2 sequence.

Castle1 was synthesised in *E. coli*. The interaction of Castle1 with sera from a poultry farm indicates that chickens vaccinated with NDV vaccines produce antibodies that are specific to the epitopes contained within Castle1. That favourably testifies that selected epitopes could be appropriate for immune stimulation in birds.

The crucial defining feature of this work is the use of relevant genotype VII sequences as a basis for recombinant protein design. In the present work, for the first time, three variants of the fragment 1 sequence were selected simultaneously to provide better coverage. Only fragment 1.1 of Castle1 interacted with sera from the poultry farm in a Western blot analysis. It could be explained by the fact that farm chickens were vaccinated with the vaccines corresponding to variant 1 of fr1. This underlines the antigenic difference between fragments and highlights the advantage of utilising the set of fragments simultaneously.

Even more importantly, our preliminary immunological study conducted on mice and chickens demonstrated that sera from animals immunised with Castle1 reacted with NDV samples belonging to two genotypes (NDV_3639_—genotype I, NDV_6081_—genotype VII). This proves the design of Castle 1 to be rational for the potential prevention of NDV infection caused by the genotypes prevailing nowadays (Table 1).

The conclusive result was that the ability of sera from Castle1-immunised mice to inhibit haemagglutination was also evaluated. HAI titres in these sera were comparable to those in sera from mice which were immunised with inactivated virus in the same manner. The favourable results of the haemagglutination inhibition test are encouraging. Given that Castle1 includes three variants of fragment 1, which together cover 69% of the analysed modern sequences (Table S2), it could be supposed as a promising basis for a potential vaccine that would be effective against a number of actual antigenic variants of NDV belonging to different genotypes.

## 4. Limitation of the Study

The main limitation of this study is that no experimental challenge study of Castle1 was performed in the target host (chickens). Within the current first stage of the antigen-development study, only a haemagglutination inhibition test using sera from mice, which are the most common first-line animal model, was performed. Moreover, the main part of the described Castle1 immunogenicity evaluation is a qualitative proof-of-concept experiment carried out on mice. Only preliminary immunogenicity studies on chicken were performed; further comprehensive experiments are required. Another limitation is that sera from chicken that recovered after infection with the genotype VII isolates with the sequences corresponding to fr1.2, fr1.3, and fr2 (or vaccinated with vaccines including the corresponding sequences) were unavailable in the current research. Fr1.2, fr1.3, and fr2 were selected primarily on the basis of a bioinformatics analysis of currently circulating isolates of NDV, and their antigenic characterisation requires additional experimental testing with the corresponding sera.

## 5. Materials and Methods

### 5.1. HN Gene Sequence Variability Analysis and Consensus Sequence Generation

To find out the distribution of NDV genotypes and the most common HN sequences for different genotypes, HN sequences collected in 2015–2026 were analysed. HN sequences with denoted genotypes were taken from the NCBI GenBank database. The consensus HN sequences for each genotype were generated using Jalview 2.11.5.2 (https://www.jalview.org, accessed on 16 January 2026) [40]. “Clustal” colouring was applied.

### 5.2. Generation of HN-Based Antigens’ Genetic Constructs

The recombinant antigen Castle1 consists of fragments corresponding to two of the NDV HN regions: 341–368 aa (fragment 1, fr1) and 239–257 aa (fragment 2, fr2). Fragment 1 is represented by three variants (fr1.1, fr1.2, and fr1.3), which differ from each other at the 347 and 362 amino acid positions. Castle1 contains two copies of each fragment (fr1.1-fr1.2-fr1.3-fr2).

The Castle1 protein gene was constructed in stages from four DNA fragments corresponding to fr1.1, fr1.2, fr1.3, and fr2. Each fragment contained a BamHI restriction site at the 5′-end and BglII and HindIII restriction sites at the 3′-end. These fragments were produced by hybridisation and elongation by the Klenow fragment (Fermentas, Vilnius, Lithuania) of a corresponding partially overlapping oligonucleotide pair (forward and reverse) (Table 5). The sequences of oligonucleotide were obtained by reverse translation *in silico* considering *E. coli* codon usage (https://www.genscript.com/tools/codon-frequency-table, accessed on 16 January 2026).

The resulting fragments were subsequently cloned into the pUC-19 vector in four restriction-ligation stages using BamHI and HindIII for both vector and insert digestion (fr.1.1) or BglII and HindIII for vector digestion and BamHI and HindIII for insert digestion (fr1.2, fr1.3 and fr2). Within the resulting construct (pUC-19-fr1.1-fr1.2-fr1.3-fr2), the fr1.1-fr1.2-fr1.3-fr2 sequence was then duplicated by digestion with BamHI and HindIII (to obtain the insert) or BglII and HindIII (to obtain vector for cloning) and following ligation. The obtained construct pUC-19-(fr1.1-fr1.2-fr1.3-fr2)2 was named pUC-Castle1. Finally, the Castle1 gene was cloned into the pQE-30 (Qiagen, Hilden, Germany) vector using the BamHI and HindIII sites (pQE-Castle1).

Constructs encoding recombinant proteins displaying each of the selected fragment separately at the C-terminus of dihydrofolate reductase (DHFR) were obtained in the pQE-40 vector (Qiagen). The pQE-40 vector encodes DHFR downstream of the His_6_-tag-coding sequence and upstream of the multi-cloning site. Fr1.1, fr1.2, fr1.3, and fr2 DNA fragments were cloned into the pQE-40 vector using the BglII and HindIII sites, resulting in the pQE-DHFR-fr1.1, pQE-DHFR-fr1.2, pQE-DHFR-fr1.3, and pQE-DHFR-fr2 plasmids. They were utilised for the expression of DHFR-fr1.1, DHFR-fr1.2, DHFR-fr1.3, and DHFR-fr2 proteins, respectively. For the unfused DHFR expression, the native pQE-40 vector was used.

### 5.3. Expression and Purification of Recombinant Proteins

*E. coli* strains SG13009 (Qiagen) and XL1-Blue (Stratagene, La Jolla, CA, USA) were employed to express Castle1. DHFR-fr1.1, DHFR-fr1.2, DHFR-fr1.3, DHFR-fr2, and DHFR were expressed in the XL1-Blue strain. Bacterial cultures were grown in 2YT medium supplemented with ampicillin (100 μg/mL) (XL1-Blue and SG13009) and kanamycin (50 μg/mL) (SG13009 only). Cultivation and harvesting were performed as described previously [26]. Purification of His_6_-tagged proteins was carried out, according to the manufacturer’s protocol (Qiagen), by Ni^2+^-NTA chromatography under denaturing conditions. Proteins were dialysed against deionised water at a 1:250 ratio for 4 h with hourly water changes. During the 4th hour, MilliQ (Simplicity UV, Merck Millipore, Darmstadt, Germany) was used for dialysis. Proteins were sterilised using 0.2 μm filters (CHROMAFIL^®^ CA-20/25(S), 729024; MACHEREY-NAGEL GmbH & Co. KG, Düren, Germany).

The theoretical molecular weights (Mr, kDa), isoelectric points (pI), and extinction coefficients (E 0.1%) of recombinant proteins were calculated using PROTPARAM from the Expert Protein Analysis System (EXPaSy) (https://web.expasy.org/protparam/, accessed on 11 April 2026) proteomics server of the Swiss Institute of Bioinformatics. Protein concentrations were determined using spectrophotometry (Nanodrop 2000, Thermo Scientific, Waltham, MA, USA) at 280 nm (E_280nm_ 0.1% Castle1 = 0.693; E_280nm_ 0.1% DHFR-fr1.1 = 1.049; E_280nm_ 0.1% DHFR-fr1.2 = 1.049; E_280nm_ 0.1% DHFR-fr1.3 = 1.047; E_280nm_ 0.1% DHFR-fr2 = 0.987; E2_80nm_ 0.1% DHFR = 1.031). Molecular weights (Mr, kDa) were calculated using the ChemiDoc^TM^ XRS+ System (Bio-Rad laboratories, Hercules, CA, USA) with Image Lab^TM^ Software 2.0.1 (Bio-Rad Laboratories, Hercules, CA, USA) by linear regression.

### 5.4. Poultry Farm Chicken Sera

Chicken serum samples were kindly provided by Chamzinskaya Poultry Farm (Sabur-Machkasy, Russia). Chickens were vaccinated according to a commercial protocol: on the first day of life—VECTORMUNE ND (Ceva-Phylaxia Veterinary Biologicals Company 1107, Budapest, Hungary), administered subcutaneously, and Nobilis^®^ ND C2 («Intervet», MSD Animal Health Russia, Moscow, Russia), administered via coarse spray; on day 13—VH vaccine (Abic Biological Laboratories Ltd., Beit Shemesh, Israel) via drinking water.

### 5.5. Enzyme-Linked Immunosorbent Assay

Titres of antibodies specific to Castle1, recombinant rotavirus protein, and NDV strain Bor-74 VGNKI (genotype I) (NDV_Bor-74_), as well as the NDV isolates NDV/chicken/Moscow/6081/2022 (GenBank ascension #PP766718.1, genotype VII) (NDV_6081_) and NDV/duck/Moscow/3639/2008 (GenBank ascension # PP795281, genotype I) (NDV_3639_) were analysed. The NDV_Bor-74_ sample was inactivated with β-propiolactone (haemagglutination activity determined before inactivation—9.5 HAU). NDV_6081_ and NDV_3639_ samples were prepared as described previously [41].

For anti-Castle1, anti-rotavirus recombinant protein, and anti-NDV_Bor-74_ antibody titre determination, 96-well microplates (Greiner Bio-One 655001, Frickenhausen, Germany) were applied. The plates were coated with 100 μL of Castle1 (10 μg/mL water solution) or with 0.1 mL of the inactivated NDV_Bor-74_ sample (1:100 (*v*/*v*) solution in PBS) and incubated overnight at +4 °C. Then, the microplates were rinsed five times with PBS containing 0.1% Tween-20 (PBST) and blocked for 2 h at room temperature with PBS containing 1% non-fat dry milk.

For anti-NDV_6081_ and anti-NDV_3639_ antibody titre determination, 96-well microplates (#9018, Corning, Corning, NY, USA) were applied. The plates were coated with 0.15 mL of the NDV_6081_ or NDV_3639_ virus samples, respectively, prepared in PBS. Then, they were incubated for 1.5 h at room temperature, rinsed five times with distilled water, and dried. Then, the microplates were subjected to UV exposure for 35 min in order to complete virus inactivation and stored at −70 °C. Before analysis, the microplates were blocked with PBS containing 1% non-fat dry milk overnight at +4 °C.

For each antigen, the analysed sera (primary antibodies) were titrated in serial 3-fold dilutions with PBS containing 1% non-fat dry milk. Microplates were incubated overnight at +4 °C and then washed five times with PBST. Anti-mouse IgG (#115-035-003) or anti-chicken IgY (#703-035-155) HRP conjugates were used at a dilution of 1/10,000. After incubation for 1 h at +37 °C (anti-mouse IgG) or 1.5 h at room temperature (anti-chicken IgY), the microplates were washed five times with PBST. TMB substrate was utilised for staining, the enzymatic reaction was stopped after 10 min by adding H_2_SO_4_ to the 1 M final concentration, and then A_450_ was measured with a «Multiscan FC» microplate photometer (Thermo Fisher, Waltham, MA, USA). A titre was defined as the reciprocal of the serum dilution at which the absorbance was equal to the mean value of the block plus three standard deviations. In the case of an A_450_ in the starting dilution being below the mean value of the block + 3SD, the serum titre was taken to be equal to the starting dilution.

### 5.6. Western Blot Analysis

The SDS-PAGE-separated samples were transferred to a nitrocellulose membrane (Sartorius, Goettingen, Germany) by wet transfer. After that, the membrane was blocked with 5% non-fat dry milk in PBST (0.01 M Tris-HCl pH 7.4, 0.15 M NaCl, 0.05% Tween-20) and treated with one of the tested sera as primary antibodies. The dilutions used in each individual experiment are indicated in the figure captions. Then, secondary anti-mouse IgG (#115-035-003) or anti-chicken IgY (#703-035-155) HRP conjugates (Jackson ImmunoResearch Laboratories, Inc., West Grove, PA, USA) were added at a dilution of 1/10,000 in PBST. The WesternBright ECL HRP substrate (Advansta Inc., San Jose, CA, USA) was used, and the signal was detected by the ChemiDoc^TM^ XRS+ gel documentation system (Bio-Rad laboratories, Hercules, CA, USA).

### 5.7. Immunisation and Sera Preparation

White outbred female mice (aged 6–8 weeks; weighing 21–22 grammes) were immunised with Castle1 (group 1, *n* = 3) or inactivated NDV_Bor-74_ (haemagglutination activity determined before inactivation—9.5 HAU) (group 2, *n* = 3). A control group of mice were immunised with recombinant rotavirus protein (obtained previously in Granovskiy et al., 2024 [30]) (group 3, *n =* 3). Each dose contained 20 µg of Castle1 (group 1) or 20 µg of the rotavirus protein (group 3) in 0.2 mL of PBS or 0.2 mL of NDV_Bor-74_ (group 2) mixed with 0.2 mL of Freund’s Adjuvant complete (primary immunisation) or incomplete (booster immunisations) (F-5881 and F-5506, Sigma-Aldrich, St. Louis, MO, USA) (group 2). Both groups were immunised intraperitoneally three times with two-week intervals. The scheme of immunisation is presented in Figure 5. Blood samples were collected after two weeks, after the third immunisation. Serum samples were prepared by centrifugation at 5000 rpm (Eppendorf MiniSpin, Eppendorf AG, Hamburg, Germany), aliquoted, and stored at −20 °C. The use of animals was approved by the ethics committee of Lomonosov Moscow State University (application #199-zh, approved during the Bioethics Commission meeting #168-d-z held on 23 January 2025).

SPF chickens (*n* = 4) were immunised with 50 µg of Castle1 in 0.13–0.14 mL of PBS mixed with an equal volume of Freund’s Adjuvant complete. Immunisations were performed twice, with a three-week interval. Blood was collected after three weeks, after the second immunisation, together with the blood samples from intact non-immune SPF chickens (*n* = 9). Blood samples were incubated for 30 min at +37 °C and overnight at +4 °C, then centrifuged for blood clot removal, aliquoted, and stored at −70 °C.

### 5.8. Haemagglutination Inhibition Assay

Evaluation of the sera haemagglutination inhibition activity was performed with the Newcastle Disease Virus HI Test Kit (FGBI “ARRIAH”, Vladimir, Russia) according to the manufacturer’s protocol. An erythrocyte suspension was prepared from the blood collected from the wing vein of SPF chicken just before the experiment. Serum samples from mice which had no immunity to NDV were used as negative controls. HAI (haemagglutination inhibition) titres were determined as the highest serum dilution, which provided total inhibition of agglutination between erythrocytes and the NDV antigen.

### 5.9. Statistical Analysis

The Wilcoxon–Mann–Whitney Test was used to compare differences between two groups. Probability values (*p* values) of less than 0.05 were considered to be significant. The statistical processing of the results was carried out using GraphPadPrism 9.1.0.

## 6. Conclusions

The rapid spread of the highly pathogenic genotype VII of NDV and the fast emergence of new virus variants characterised by amino acid substitutions in key neutralising epitopes indicate the need for a vaccine that would be effective primarily against currently circulating highly pathogenic NDV. The recombinant antigen Castle1 that simultaneously includes three variants of HN antigenic fragment 1 (341–368 a.a.) and fragment 2 (239–257 a.a.) was designed for the first time in the present study. The utilised fragment 1 variants correspond to the sequences of genotype VII isolates currently dominating worldwide. Preliminary immunological evaluation showed that Castle1 reliably interacted with poultry farm chicken sera which are immune to two viral isolates belonging to the genotypes I and VII. It proves the reasonable selection of antigenic fragments comprising Castle1. The antigenic difference between fragment 1 variants included in Castle1 was demonstrated by Western blot. This underlines the importance of including different sequences of fragment 1 to expand antigenic coverage. Anti-Castle1 antibodies bound NDV_Bor-74_, NDV_3639_ (genotype I), and NDV_6081_ (genotype VII) in ELISA and had haemagglutination inhibition activity. Therefore, Castle1 could be considered a promising antigen for the further development of a vaccine focusing on currently prevalent NDV isolates.

## Figures and Tables

**Table 1:** Genotype distribution of NDV isolates collected 2015–2025.

	Class I	Class II									
Genotype	1	I	II	III	IV	V	VI	VII	XII	XIII	XXI
Number of isolates	123	48	26	2	2	39	84	341	16	16	15

**Table 2:** ELISA titres and Western blot results summary of sera from poultry farm.

	ELISA Titre Antigen			Western Blot Antigen		
Serum	Castle1	NDV_3639_	NDV_6081_	NDV_Bor-74_	Castle1	DHFR-fr1.1
7 m #3	656,531	146,170	79,515	+	+	−
1 yr #13	600,987	619,784	78,820	+	+	+
7 m #6	199,521	541,588	≥243,000 **	+	+	+
1 yr #5	186,301	1,305,698	1,049,994	+	−	−
1 yr #9	166,989	1,835,989	421,154	+	+	+
2 d #1	141,030	651,548	706,092	+	+	+

**—The exact titre is unknown due to an experimental artefact and the limited quantity of the serum. 1 yr—1-year-old group. 7 m—7-month-old group. 2 d—2-day-old group. The sign “#” indicates a number of a serum.

**Table 3:** ELISA titres summary results of mice sera after immunisation with Castle1 or NDV_Bor-74_.

Immunogen	Serum	Antigen in ELISA				
		Castle1	NDV_Bor-74_	NDV_3639_	NDV_6081_	Rotavirus Protein
NDV_Bor-74_	Bor#1	515,943	2,645,227	662,700	203,119	3348
	Bor#2	1,891,791	26,721,179	>729,000	>729,000	729
	Bor#3	1,553,941	2,375,398	616,067	190,205	814
Castle1	Cas#1	26,352,781	2,266,194	>729,000	111,567	546
	Cas#2	8,177,917	896,172	206,558	80,752	2033
	Cas#3	1,041,203	941,181	241,890	113,751	3991
Rotavirus protein (negative control)	Rota#1	1266	1863	1287	830	>21,870,000
	Rota#2	3345	1178	1004	692	2,292,171
	Rota#3	1172	741	2427	300	1,343,036

The sign “#” indicates a number of a serum.

**Table 4:** Haemagglutination inhibition activity test results for mice sera after immunisation with Castle1 or NDV_Bor-74_.

Immunogen	Serum	Mean log_2_ (HAI Titre) (*n* = 3)
NDV_Bor-74_	Bor#1	7.33
	Bor#2	9
	Bor#3	7.66
Castle1	Cas#1	9.66
	Cas#2	6.33
	Cas#3	7

The sign “#” indicates a number of a serum.

**Table 5:** Oligonucleotides’ sequences for Castle1 genetic construct generation.

Name	Sequence (5′ to 3′)
fr1.1 Fwd	acat*ggatcc*aacaacaccagcccggat**gaa**caggattatcagattcgtatggcgaaatctagctacaaac
fr1.1 Rev	atac*aagctt*accgcc*agatct*tttaccgccaaagcg**gcc**cggtttgtagctagatttcgccata
fr1.2 Fwd	acat*ggatcc*aacaacaccagcccggac**aaa**caggattatcagatccgtatggcgaaatctagctacaaac
fr1.2 Rev	atac*aagctt*accgcc*agatct*tttgccaccgaagcg**cgc**cggtttgtagctagatttcgccata
fr1.3 Fwd	acat*ggatcc*aacaacaccagcccggat**cag**caggactatcagattcgtatggcgaaatctagctacaaac
fr1.3 Rev	atac*aagctt*accgcc*agatct*tttaccgccaaagcg**ttt**cggtttgtagctagatttcgccata
fr2 Fwd	acat*ggatcc*gtgagcgcgaccccgctgggtagcgatatgctgagctcta
fr2 Rev	atac*aagctt*accgcc*agatct*ggtgccggtcactttagagctcagcatatcgctacc

Restriction sites BamHI, BgIII, and HindIII are italicised and highlighted in yellow, magenta, and cyan, respectively. Codons corresponding to amino acids that diversify fragments 1.1, 1.2, and 1.3 are highlighted in bold and coloured in blue, green, and brown, respectively. Sequences overlapping in each primers pair are underlined.

## Data Availability

All the relevant data are provided in this paper and its Supplementary Materials.

## Supplementary Materials

The following supporting information can be downloaded at https://www.mdpi.com/article/10.3390/ijms27198673/s1.

## Abbreviations

The following abbreviations are used in this manuscript:

DHFR	Dihydrofolate reductase
ECL	Enhanced chemiluminescence
ELISA	Enzyme-linked immunosorbent assay
F-protein	Fusion protein
Fr1	Fragment 1
Fr2	Fragment 2
GST	Glutathione S-transferase
HAI	Haemagglutination inhibition
HAU	Haemagglutinating Unit
HN	Haemagglutinin-neuraminidase
HRP	Horseradish peroxidase
MHC	Major Histocompatibility Complex
MW	Molecular weights
NDV	Newcastle disease virus
Ni^2+^-NTA	Ni^2+^-nitrilotriacetic acid
PBS	Phosphate-buffered saline
PBST	Phosphate-buffered saline with Tween 20
PDB	Protein Data Bank
pI	Isoelectric point
rF	Recombinant fusion protein
rHN	Recombinant haemagglutinin-neuraminidase
SDS-PAGE	Sodium dodecyl sulphate polyacrylamide gel electrophoresis
SPF	Specific pathogens free
TMB	3,3′,5,5′-tetramethylbenzidine
USDA	United States Department of Agriculture

## References

[1] Poultryworld.Net. https://www.poultryworld.net/health-nutrition/health/newcastle-disease-spreads-further-in-europe.

[2] Kondakova O.A., Agranovsky A.A., Ryabchevskaya E.M., Umarova E.P., Granovskiy D.L., Toropov S.E., Evtushenko E.A., Nikitin N.A., Karpova O.V. (2025). Genetic Diversity of Newcastle Disease Virus and Its Implications for Vaccine Development. Vet. Sci..

[3] Chambers P., Nesbit M., Yusoff K., Millar N.S., Samson A.C.R., Emmerson P.T. (1988). Location of a Neutralizing Epitope for the Haemagglutinin-Neuraminidase Glycoprotein of Newcastle Disease Virus. J. Gen. Virol..

[4] Mayahi V., Esmaelizad M. (2017). Identification of Novel E347Q and G362K Amino Acid Substitutions in HN Neutralization Epitope and Major Antigenic Difference in Novel Sub-Genotype VIIj Isolates. Acta Virol..

[5] Guseva N.A., Kolosov S.N., Zinyakov N.G., Kozlov A.A., Shcherbakova L.O., Chvala I.A., Andriyasov A.V., Yin R., Andreychuk D.B., Chvala I.A. (2025). Subgenotype VII.1.1 Newcastle Disease Virus Evolution and Spread in the Russian Federation in 2019–2023. Viruses.

[6] Dimitrov K.M., Abolnik C., Afonso C.L., Albina E., Bahl J., Berg M., Briand F.-X., Brown I.H., Choi K.-S., Chvala I. (2019). Updated Unified Phylogenetic Classification System and Revised Nomenclature for Newcastle Disease Virus. Infect. Genet. Evol..

[7] Dimitrov K.M., Ramey A.M., Qiu X., Bahl J., Afonso C.L. (2016). Temporal, Geographic, and Host Distribution of Avian Paramyxovirus 1 (Newcastle Disease Virus). Infect. Genet. Evol..

[8] WOAH Newcastle Disease (Infection with Newcastle Disease Virus). World Organisation for Animal Health. 2019. https://www.woah.org/fileadmin/Home/eng/Health_standards/tahm/3.03.10_NEWCASTLE_DIS.pdf.

[9] Alazawy A.K., Al Ajeeli K.S. (2020). Isolation and Molecular Identification of Wild Newcastle Disease Virus Isolated from Broiler Farms of Diyala Province, Iraq. Vet. World.

[10] Mariappan A.K., Munusamy P., Kumar D., Latheef S.K., Singh S.D., Singh R., Dhama K. (2018). Pathological and Molecular Investigation of Velogenic Viscerotropic Newcastle Disease Outbreak in a Vaccinated Chicken Flocks. VirusDisease.

[11] Gowthaman V., Ganesan V., Gopala Krishna Murthy T.R., Nair S., Yegavinti N., Saraswathy P.V., Suresh Kumar G., Udhayavel S., Senthilvel K., Subbiah M. (2019). Molecular Phylogenetics of Newcastle Disease Viruses Isolated from Vaccinated Flocks during Outbreaks in Southern India Reveals Circulation of a Novel Sub-genotype. Transbound. Emerg. Dis..

[12] Lee Y.-J., Sung H.-W., Choi J.-G., Lee E.-K., Yoon H., Kim J.-H., Song C.-S. (2008). Protection of Chickens from Newcastle Disease with a Recombinant Baculovirus Subunit Vaccine Expressing the Fusion and Hemagglutinin-Neuraminidase Proteins. J. Vet. Sci..

[13] Yusibov V., Streatfield S.J., Kushnir N. (2011). Clinical Development of Plant-Produced Recombinant Pharmaceuticals: Vaccines, Antibodies and Beyond. Hum. Vaccines.

[14] Lee J.-W., Huang J.-P., Hong L.-S., Shu S.-F., Yu C., Chu C.-Y. (2010). Prokaryotic Recombinant Hemagglutinin-Neuraminidase Protein Enhances the Humoral Response and Efficacy of Commercial Newcastle Disease Vaccines in Chickens. Avian Dis..

[15] De Groot A.S., Moise L., McMurry J.A., Martin W., Falus A. (2009). Epitope-Based Immunome-Derived Vaccines: A Strategy for Improved Design and Safety. Clinical Applications of Immunomics.

[16] Li J., Ju Y., Jiang M., Li S., Yang X.-Y. (2025). Epitope-Based Vaccines: The Next Generation of Promising Vaccines Against Bacterial Infection. Vaccines.

[17] Toyoda T., Gotoh B., Sakaguchi T., Kida H., Nagai Y. (1988). Identification of Amino Acids Relevant to Three Antigenic Determinants on the Fusion Protein of Newcastle Disease Virus That Are Involved in Fusion Inhibition and Neutralization. J. Virol..

[18] Yusoff K., Nesbit M., McCartney H., Meulemans G., Alexander D.J., Collins M.S., Emmerson P.T., Samson A.C.R. (1989). Location of Neutralizing Epitopes on the Fusion Protein of Newcastle Disease Virus Strain Beaudette C. J. Gen. Virol..

[19] Cho S.-H., Kwon H.-J., Kim T.-E., Kim J.-H., Yoo H.-S., Kim S.-J. (2008). Variation of a Newcastle Disease Virus Hemagglutinin-Neuraminidase Linear Epitope. J. Clin. Microbiol..

[20] Hu S., Wang T., Liu Y., Meng C., Wang X., Wu Y., Liu X. (2010). Identification of a Variable Epitope on the Newcastle Disease Virus Hemagglutinin–Neuraminidase Protein. Vet. Microbiol..

[21] Zhu J., Hu S., Xu H., Liu J., Zhao Z., Wang X., Liu X. (2016). Characterization of Virulent Newcastle Disease Viruses from Vaccinated Chicken Flocks in Eastern China. BMC Vet. Res..

[22] Jin Z., Wei Q., Bi Y., Li Y., Huo N., Mou S., Wang W., Liu H., Yang Z., Chen H. (2021). Identification of a Potential Neutralizing Linear Epitope of Hemagglutinin-Neuraminidase in Newcastle Disease Virus. Virol. J..

[23] Scanlon D.B., Corino G.L., Shiell B.J., Della-Porta A.J., Manvell R.J., Alexander D.J., Hodder A.N., Gorman J.J. (1999). Pathotyping Isolates of Newcastle Disease Virus Using Antipeptide Antibodies to Pathotype-Specific Regions of Their Fusion and Hemagglutinin-Neuraminidase Proteins. Arch. Virol..

[24] Mayahi V., Esmaelizad M., Ganjalikhany M.R. (2020). Development of Avian Avulavirus 1 Epitope-Based Vaccine Pattern Based on Epitope Prediction and Molecular Docking Analysis: An Immunoinformatic Approach. Int. J. Pept. Res. Ther..

[25] Tavassoli A., Soleymani S., Housaindokht M.R. (2024). Nucleotide Sequence Characterization, Amino Acid Variations and 3D Structural Analysis of HN Protein of the NDV VIId Genotype. Vet. Med. Sci..

[26] Berman H.M. (2000). The Protein Data Bank. Nucleic Acids Res..

[27] Zaitsev V., Von Itzstein M., Groves D., Kiefel M., Takimoto T., Portner A., Taylor G. (2004). Second Sialic Acid Binding Site in Newcastle Disease Virus Hemagglutinin-Neuraminidase: Implications for Fusion. J. Virol..

[28] Kovalenko A.O., Ryabchevskaya E.M., Evtushenko E.A., Manukhova T.I., Kondakova O.A., Ivanov P.A., Arkhipenko M.V., Gushchin V.A., Nikitin N.A., Karpova O.V. (2022). Vaccine Candidate Against COVID-19 Based on Structurally Modified Plant Virus as an Adjuvant. Front. Microbiol..

[29] Ryabchevskaya E.M., Granovskiy D.L., Evtushenko E.A., Ivanov P.A., Kondakova O.A., Nikitin N.A., Karpova O.V. (2022). Designing Stable Bacillus Anthracis Antigens with a View to Recombinant Anthrax Vaccine Development. Pharmaceutics.

[30] Granovskiy D.L., Khudainazarova N.S., Evtushenko E.A., Ryabchevskaya E.M., Kondakova O.A., Arkhipenko M.V., Kovrizhko M.V., Kolpakova E.P., Tverdokhlebova T.I., Nikitin N.A. (2024). Novel Universal Recombinant Rotavirus A Vaccine Candidate: Evaluation of Immunological Properties. Viruses.

[31] Jamour P., Shafaati M., Gigloo M.G., Mehrabzadeh R.S., Mohammadi T., Lotfinia M., Majidi S. (2025). Serum Reactivity Analysis with Inactivated GVII-Matched Vaccine—Payavax G79^®^: Comparison of B-Cell Epitopes in NDV-Vaccine Strains. Biologicals.

[32] Ji J., Dong X., Liu X., Ding M., Lin Y., Zhang Y., Zhang W., Lei B., Yuan W., Zhao K. (2025). Designing a Novel Multi-Epitope Trivalent Vaccine Against NDV, AIV and FAdV-4 Based on Immunoinformatics Approaches. Microorganisms.

[33] Motamedi M.J., Shahsavandi S., Amani J., Kazemi R., Takrim S., Jafari M., Hataf Salmanian A. (2018). Immunogenicity of the Multi-Epitopic Recombinant Glycoproteins of Newcastle Disease Virus: Implications for the Serodiagnosis Applications. Iran. J. Biotechnol..

[34] Mozafari A., Rahmani M., Yasini Nasab Y., Shahsavandi S., Jafari M., Salmanian A.H. (2024). The Heterologous Expression of Novel Recombinant Protein Composed of HN and F Moieties of Newcastle Disease Virus and Immunogenicity Evaluation in Mouse Model. Iran. J. Microbiol..

[35] Chang Z., Dong X., Guan Z., Lu K., Chen X., Wei X., Guo H., Dang R., Wang J., Wang X. (2023). Antigenic Variation in Hemagglutinin-Neuraminidase of Newcastle Disease Virus Isolated from Tibet, China. Vet. Microbiol..

[36] Zheng B., Graham F.L., Johnson D.C., Hanke T., McDermott M.R., Prevec L. (1993). Immunogenicity in Mice of Tandem Repeats of an Epitope from Herpes Simplex gD Protein When Expressed by Recombinant Adenovirus Vectors. Vaccine.

[37] Trifonova E.A., Zenin V.A., Nikitin N.A., Yurkova M.S., Ryabchevskaya E.M., Putlyaev E.V., Donchenko E.K., Kondakova O.A., Fedorov A.N., Atabekov J.G. (2017). Study of Rubella Candidate Vaccine Based on a Structurally Modified Plant Virus. Antivir. Res..

[38] Kondakova O.A., Ivanov P.A., Baranov O.A., Ryabchevskaya E.M., Arkhipenko M.V., Skurat E.V., Evtushenko E.A., Nikitin N.A., Karpova O.V. (2021). Novel Antigen Panel for Modern Broad-Spectrum Recombinant Rotavirus A Vaccine. Clin. Exp. Vaccine Res..

[39] Tarigan S., Dharmayanti N.L.P.I., Sugiartanti D., Putri R., Andriani, Nuradji H., Robinson M., Wiendayanthi N., Djufri F. (2023). Characterization of Two Linear Epitopes SARS CoV-2 Spike Protein Formulated in Tandem Repeat. PLoS ONE.

[40] Waterhouse A.M., Procter J.B., Martin D.M.A., Clamp M., Barton G.J. (2009). Jalview Version 2—A Multiple Sequence Alignment Editor and Analysis Workbench. Bioinformatics.

[41] Boravleva E., Treshchalina A., Gordeeva D., Gambaryan A. (2024). Development of an Inexpensive and Simple Test System for the Differential Detection of Avian Influenza Viruses and Avian Paramyxoviruses in Environmental Monitoring. J. Res. Vet. Sci..

