# Supplementary material for: Recombinant Antigen for Newcastle Disease Virus Vaccine Focusing on Currently Circulating Virus Variants
Source: Int J Mol Sci. 2026 Sep 28;27(19):8673. doi: 10.3390/ijms27198673 (PMC13664881; doi:10.3390/ijms27198673)
Supplement: Supplementary file 1 [file ijms-27-08673-s001.zip › Supplementary materials ijms-4461252.pdf]

International Journal of
Molecular Sciences

MDPI

Supplementary Materials: Recombinant Antigen for Newcastle Disease Virus Vaccine Focusing on Currently Circulating Virus Variants

Elizaveta P. Umarova [1], Ekaterina M. Ryabchevskaya [1,*], Dmitriy L. Granovskiy [1], Ekaterina A. Evtushenko [1], Angelina O. Kovalenko [1], Elizaveta Yu. Boravleva [2], Aleksandra S. Gambaryan [2], Irina N. Matveeva [3], Nikolai A. Nikitin [1] and Olga V. Karpova [1]

Table S2. Frequencies of various 347and 362 aa pairs within collected after 2015 NDV class II isolates with known genotypes

Class II Genotype I (46 sequences)				
347 aa		362 aa		pair %
E	46	G	46	100,00%

Class II Genotype II (26 sequences)				
347 aa		362 aa		pair %
E	26	G	25	96,15%
G			1	3,85%

Class II Genotype V (39 sequences)				
347 aa		362 aa		pair %
E	30	G	39	76,92%
G	5			12,82%
K	4			10,26%

Class II Genotype XII (16 sequences)				
347 aa		362 aa		pair %
D	13	R	13	81,25%
E	3	G	3	18,75%

Class II Genotype VI (84 sequences)				
347 aa		362 aa		pair %
G	82	G	84	97,62%
E	2			2,38%

Class II Genotype XIII (16 sequences)				
347 aa		362 aa		pair %
E	12	R	7	43,75%
		G	5	31,25%
G	4	G	4	25,00%

Class II Genotype VII (316 sequences)				
347 aa		362 aa		pair %
Q	142	K	142	44,94%
K	91	A	61	19,30%
		G	25	7,91%
		Q	5	1,58%
E	81	G	55	17,41%
		Q	24	7,59%
		E	2	0,63%
R	2	A	2	0,63%

Class II Genotype XXI (15 sequences)				
347 aa		362 aa		pair %
E	15	G	15	100,00%

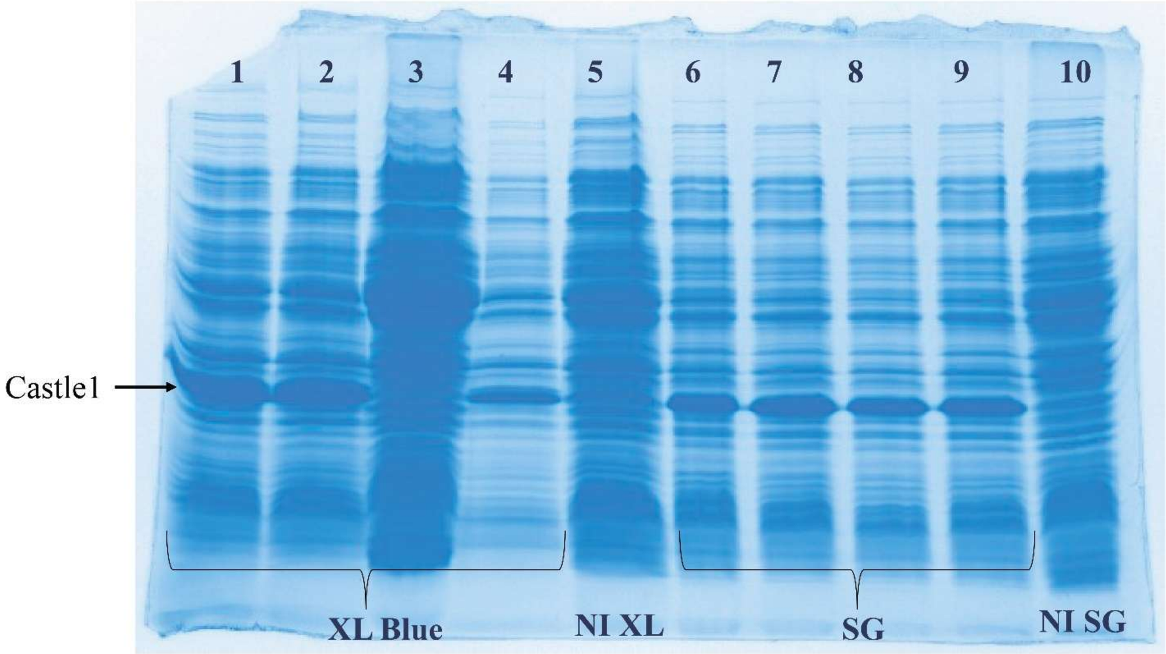

Figure S1. The Castle1 expression efficacy in *E. coli* strains XL1-Blue and SG13009
1-4 – induced *E. coli* XL1-Blue cell clones; 5 – *E. coli* XL1-Blue cell clone before induction; 6-9 – induced *E. coli* SG13009 cell clones; 10 –*E. coli* SG13009 cell clone before induction.

A

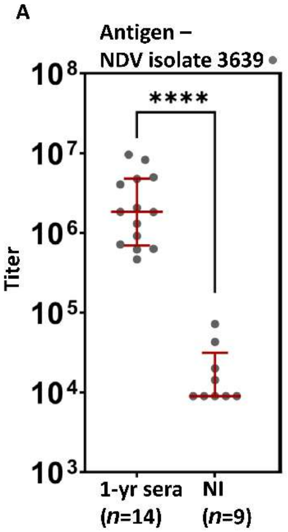

	NI	1-yr sera
1	9000	9,588,084
2	42,919	915,814
3	9000	8,242,327
4	9000	4,747,628
5	14,333	1,305,698
6	9000	718,871
7	9000	465,742
8	72,210	4,981,449
9	20,046	1,835,989
10		1,839,843
11		630,705
12		4,047,496
13		619,784
14		2,062,763
median	9000	1,837,916

B

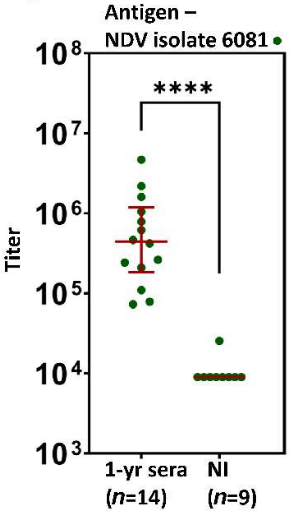

	NI	1-yr sera
1	9000	4,674,833
2	25,472	242,833
3	9000	2,185,576
4	9000	1,601,094
5	9000	1,049,994
6	9000	262,385
7	9000	72,897
8	9000	787,154
9	9000	421,154
10		465,132
11		109,530
12		616,928
13		78,820
14		209,095
median	9000	443,143

Figure S2. Titres of antibodies specific to different isolates of NDV in sera from poultry farm

(A) Titres of antibodies to NDV_{3639}

(B) Titres of antibodies to NDV_{6081}

Sera titres were evaluated using indirect ELISA. The group of poultry farm sera was compared with the non-immune group (intact SPF chickens). *p*-values were calculated using the Mann–Whitney Test. ●, IgY titres of individual chicken; NI, non-immunised group; ****p <0,0001; —, median. Error bars represent the interquartile range. When A_{450} was lower than the background signal + 3 SD the serum titre was taken as 9000 (due to start dilution was 1:9000).

NDV_{3639} - NDV/duck/Moscow/3639/2008 isolate

NDV_{6081} - NDV/chicken/Moscow/6081/2022 isolate

1-yr sera, 1-year-old chicken sera group

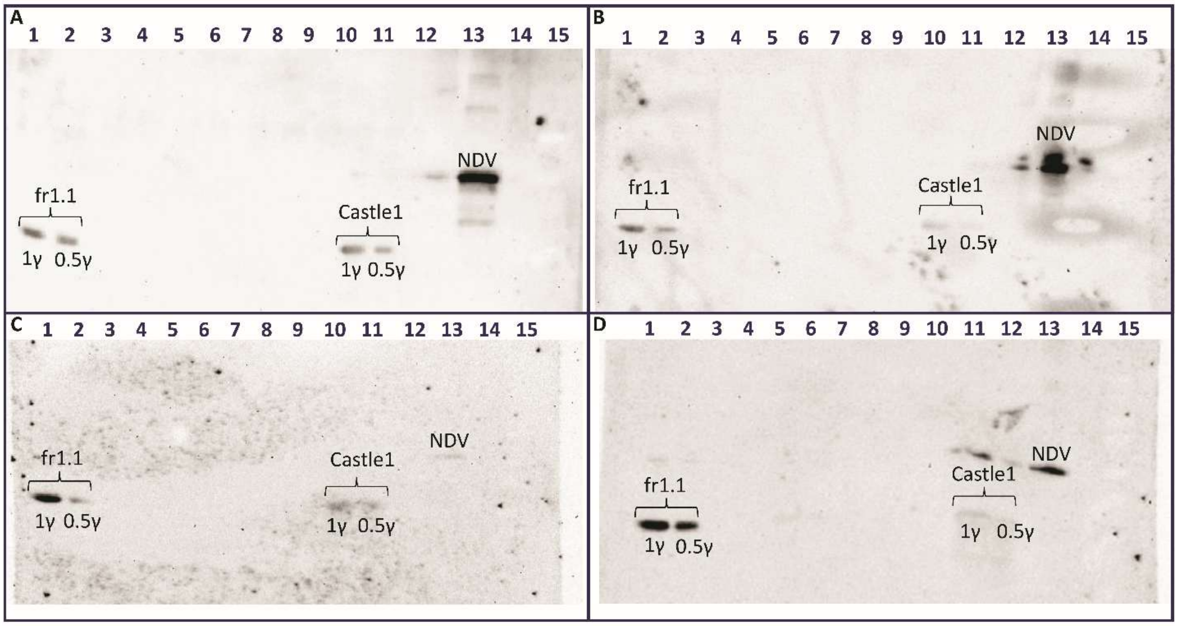

Figure S3. Study of the antigenic diversity of Castle1-composing fragments

Western blot analysis with primary chicken's sera from poultry farm and secondary HRP-conjugated antibodies (1:10000)

(A) 1-year-old group #13 serum (1:3000)

(B) 1-year-old group #9 serum (1:6000)

(C) 2-day-old group #1 serum (1:3000)

(D) 7-month-old group #6 serum (1:3000)

(A-C) 1 (1γ), 2 (0,5γ) – fr1.1; 3 (1γ), 4 (0,5γ) – fr1.2; 5 (1γ), 6 (0,5γ) – fr1.3; 7 (1γ), 8 (0,5γ) – fr2; 9 (1γ) – DHFR (negative control); 10 (1γ), 11 (0,5γ) – Castle1; 13 – NDV (strain Bor-74) (positive control); 14 (1γ) – recombinant His_6-containing Coronavirus antigen* expressed in *E. coli* (negative control); 15 – protein ladder.

(D) 1 (1γ), 2 (0,5γ) – fr1.1; 3 (1γ), 4 (0,5γ) – fr1.2; 5 (1γ), 6 (0,5γ) – fr1.3; 7 (1γ), 8 (0,5γ) – fr2; 9 (1γ) – DHFR (negative control); 11 (1γ), 12 (0,5γ) – Castle1; 13 – NDV (strain Bor-74) (positive control); 14 (1γ) – recombinant His_6-containing Coronavirus antigen* expressed in *E. coli* (negative control); 15 – protein ladder. The quantity of protein loaded into a slot is indicated: 1γ = 1 μg; 0,5γ = 0,5 μg.

* - obtained previously in Kovalenko et al., 2022 [28]

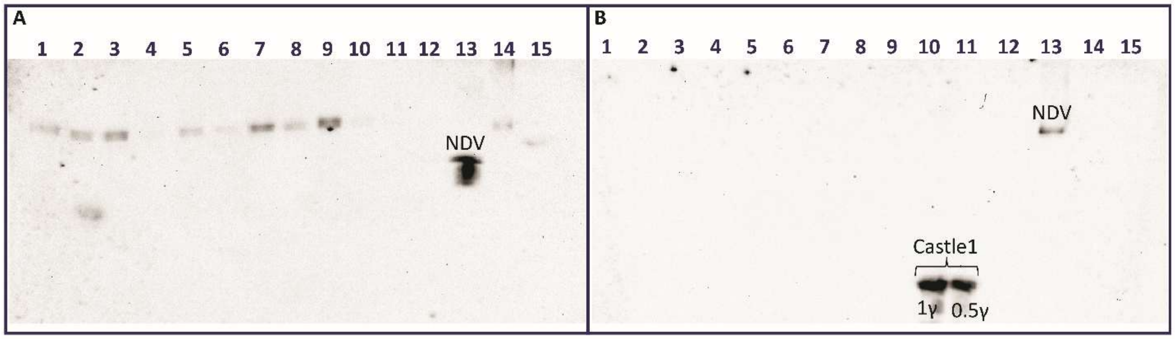

Figure S4. Study of the antigenic diversity of Castle1-composing fragments

Western blot analysis with primary chicken's sera from poultry farm and secondary HRP-conjugated antibodies (1:10000)

(A) 1-year-old group #5 serum (1:3000)

(B) 7-month-old group #3 serum (1:3000)

(A) 1 (1γ), 2 (0,5γ) – fr1.1; 3 (1γ), 4 (0,5γ) – fr1.2; 5 (1γ), 6 (0,5γ) – fr1.3; 7 (1γ), 8 (0,5γ) – fr2; 9 (1γ) – DHFR (negative control); 11 (1γ), 12 (0,5γ) – Castle1; 13 – NDV (strain Bor-74) (positive control); 14 (1γ) – recombinant His_6-containing Coronavirus antigen* expressed in *E. coli* (negative control); 15 – protein ladder.

(B) 1 (1γ), 2 (0,5γ) – fr1.1; 3 (1γ), 4 (0,5γ) – fr1.2; 5 (1γ), 6 (0,5γ) – fr1.3; 7 (1γ), 8 (0,5γ) – fr2; 9 (1γ) – DHFR (negative control); 10 (1γ), 11 (0,5γ) – Castle1; 13 – NDV (strain Bor-74) (positive control); 14 (1γ) – recombinant His_6-containing Coronavirus antigen* expressed in *E. coli* (negative control); 15 – protein ladder. The quantity of protein loaded into a slot is indicated: 1γ = 1 μg; 0,5γ = 0,5 μg.

* - obtained previously in Kovalenko et al., 2022 [28]

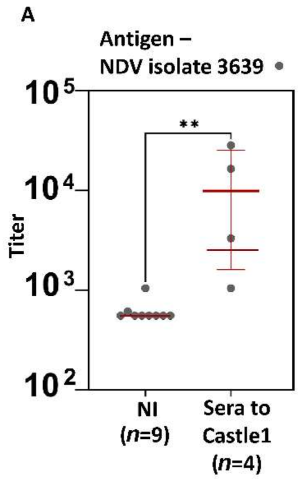

	NI	Sera to Castle1
1	612	28,338
2	1047	3308
3	556	1046
4	556	16,478
5	556	
6	556	
7	556	
8	556	
9	556	
median	556	9893

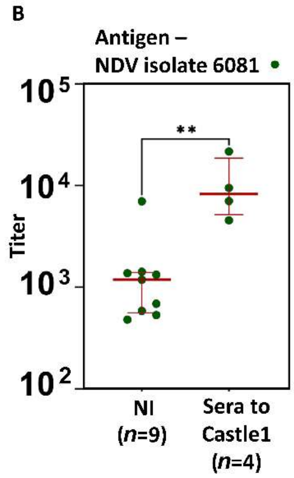

	NI	Sera to Castle1
1	1186	7052
2	7007	9492
3	1327	4550
4	1375	21,651
5	533	
6	689	
7	586	
8	1419	
9	480	
median	1186	8272

Figure S5. Titres of antibodies specific to different isolates of NDV in sera from Castle1-immunised chickens

(A) Titres of antibodies to NDV_{3639}

(B) Titres of antibodies to NDV_{6081}

Sera titres were evaluated using indirect ELISA. The group of sera form chickens immunised with Castle1 was compared with the group of sera from intact non-immune group. *p*-values were calculated using the Mann–Whitney Test. ●, IgY titres of individual chicken; NI, non-immunised group; $^{**}p$ <0,01; ▬ , median. Error bars represent the interquartile range. In case of the evaluation of antibody titres to NDV_{3639} (A) the start dilution was 1:556, therefore when A_{450} was lower than the background signal + 3 SD the serum titre was taken as 556.

NDV_{3639} - NDV/duck/Moscow/3639/2008 isolate

NDV_{6081} - NDV/chicken/Moscow/6081/2022 isolate
